# Seroprevalence of Hepatitis B Among Healthcare Workers in Asia and Africa and Its Association With Their Knowledge and Awareness: A Systematic Review and Meta-Analysis

**DOI:** 10.3389/fpubh.2022.859350

**Published:** 2022-04-28

**Authors:** Nur Hasnah Maamor, Nor Asiah Muhamad, Nor Soleha Mohd Dali, Mohd Hatta Abdul Mutalip, Fatin Norhasny Leman, Tahir Aris, Nai Ming Lai, Muhammad Radzi Abu Hassan

**Affiliations:** ^1^Sector for Evidence-Based Healthcare, National Institutes of Health, Ministry of Health, Setia Alam, Malaysia; ^2^Institute for Medical Research, National Institutes of Health, Ministry of Health, Setia Alam, Malaysia; ^3^Institute for Public Health, National Institute of Health, Ministry of Health, Setia Alam, Malaysia; ^4^School of Medicine, Taylor's University, Subang Jaya, Malaysia; ^5^Department of Medicine, Sultanah Bahiyah Hospital, Ministry of Health, Alor Setar, Malaysia; ^6^Clinical Research Centre, Sultanah Bahiyah Hospital, Ministry of Health, Alor Setar, Malaysia

**Keywords:** hepatitis B virus, seroprevalence, prevalence, infection, healthcare workers, knowledge, awareness, epidemiology

## Abstract

**Introduction:**

The hepatitis B virus (HBV) is a blood-borne virus that can be transmitted by percutaneous and mucocutaneous contact with infected bodily fluid. Healthcare workers (HCWs) are more exposed to HBV infection. They must have a thorough understanding of HBV infection since they can contract and spread the virus. In this study, we systematically reviewed all published evidence on the seroprevalence of Hepatitis B virus (HBV) infection among HCWs. and synthesize evidence on the association between knowledge and awareness with HBV infection.

**Methods:**

We searched PubMed, EMBASE, Cochrane Library and Scopus for studies reporting on HBV seroprevalence from January 1997 to September 2021 among healthcare workers. We used random-effects meta-analyses to estimate the pool prevalence of HBV infection.

**Results:**

We identified 25 studies that met our inclusion criteria, with data on 10,043 adults from 11 countries and two regions: Africa and Asia. The overall seroprevalence of HBV was 5.0% (95% confidence interval [CI] 3.6%), with Africa reporting higher estimates (5.0%, 95% CI 3.7%) than Asia population (4.0%, 95% CI 1.9%). The highest pooled prevalence estimate in African countries came from studies published in the Cameroon region (8.0%, 95% CI 5–10%) while the lowest came from Ethiopia (4.0%, 95% CI 2.6%). The overall seroprevalence estimates in the African population were significantly higher than those in the Asian group. Studies in Africa found that the average knowledge and seroprevalence were 1.4% and 11.0%, respectively where, eight studies (53.3%) reported good knowledge and seven studies (46.7%) reported average knowledge. In Asia, two studies (40.0%) reported good knowledge, one study (20.0%) reporting average knowledge, and two studies (40.0%) reporting poor knowledge. African studies demonstrated good knowledge despite the fact that their HBV infection rate was higher than 6.7%.

**Conclusion:**

Africa and Asia have the highest seroprevalence of HBV infection. Improving the comparability of epidemiological and clinical studies constitutes an important step forward. More high-quality data is needed to improve the precision of burden estimates.

**Systematic Review Registration:**

PROSPERO CRD42021279905.

## Introduction

The Hepatitis B virus (HBV) is a bloodborne virus that has become a major global public health concern. HBV, which belongs to the *Hepadnaviridae* family, has only one known natural host: humans. The virus enters the liver through the bloodstream and replicates in the tissue of the liver (1). Acute hepatitis B infection causes inflammation and jaundice in the liver, while chronic hepatitis B infection can lead to potentially fatal diseases such as liver cirrhosis and hepatocellular carcinoma (2). Globally, HBV infected over 2 billion individuals with 250 million of them suffering from chronic HBV infection (3). According to the World Health Organization (WHO), 325 million people are infected with HBV, with the African and Western Pacific regions having the highest rates of HBV infection at 68% (4), and approximately 900,000 people dying from HBV each year (5). Hepatitis B is most common in Sub-Saharan Africa and Southeast Asia (8.0–10.0%). This is followed by Eastern and Southern Europe, the Middle East, and Japan (2.0-7.0%), and the United States and Northern Europe (0.5–2.0%) (6–8). Furthermore, it is estimated that 40% of the healthcare workers (HCWs) are infected with HBV infections in the developing countries (9).

Healthcare workers are four times more likely to be infected with HBV compared to the general population (10). This may be due to a lack of compliance with infection control recommendations from established guidelines such as the Center for Disease Control and Prevention (CDC) (11). Handwashing, glove use, and correct disposal of sharp instruments are all part of the CDC's recommended precaution, which is aimed to prevent the spread of blood-borne infections like HBV (12). In the case of HBV infection, knowledge includes information gathering, experience, skill, and disease prevention strategies (13, 14). A lack of understanding among HCWs in both low- and middle-income countries leads to low adherence to safety measures, aggravating the HBV situation (15). A better understanding of HBV infection is essential to reduce the rate of infection among HCWs in the healthcare context (16). Knowledge is usually assessed to investigate how far the community know the concepts of disease including causes and symptoms of disease. Attitude is defined as a product of a complex interaction on values, feelings and beliefs (17). Practice is defined as an action of the habitual community to prevent the disease (18). Awareness is the knowledgeable person being conscious and behavior under the receiving in taxonomy of affective domain (19).

Although HCWs are more aware of Hepatitis B, several countries lack a comprehensive grasp of the disease biology, transmission methods, risk of transmission, clinical characteristics, and vaccination availability (20). Hepatitis B virus seroprevalence among HCWs was reported in a prior study by Mahamat et al. (21), but there was no relationship study between seroprevalence and knowledge or awareness (21). Hepatitis B awareness is lower among HCWs in developing countries, which is linked to poorer preventive attitudes, including lower Hepatitis B vaccine coverage (22). On the other hand, the prevalence of HBV infection fluctuates and is influenced by a variety of factors including geographical region, host factor, and environmental or behavioral factors. The low prevalence of HBV in Europe, for example, may be attributed to the high standard of living there (10). As a result, Hepatitis B prevalence can also predict the risk factors for HBV transmission, such as injections, occupational injuries, body tattoos, and a history of not being been vaccinated, among others (23). Therefore, this study aimed to determine the pooled prevalence of hepatitis B infection among HCWs and to compare the pooled prevalence of HBV infection across different regions. We also compiled data to determine the association between seroprevalence and level of knowledge or awareness on HBV infection.

## Materials and Methods

We conducted a systematic review by following the Preferred Reporting Items for Systematic Reviews and Meta-Analyses (PRISMA) (24) and Guidelines for Accurate and Transparent Health Estimates Reporting (GATHER) statement (25). The PROSPERO registration number is CRD42021279905. Our primary outcome was the seroprevalence of HBV infection among HCWs. Our secondary outcome was the estimation of knowledge and awareness attributable to HBV infection.

### Search Strategy

A comprehensive systematic literature search was conducted in electronic databases (PubMed, EMBASE, Cochrane Library, and Scopus) to identify relevant studies from inception to September 2021. A search strategy was developed for PubMed (Supplementary Table 1) and adapted for use in the other databases. Other electronic search was performed in the WHO International Clinical Trials Registry Platform (ICTRP). In the search, key words and equivalent Medical Subject Heading (MeSH) phrases were combined when applicable, with no language or publication year restrictions. Specific search terms are as follows: “hepatitis B” OR “HBV” OR “hepatitis B virus” AND “etiology” OR “etiology” OR “prevalence” OR “epidemiology” OR “infection” AND “healthcare workers” OR “healthcare” OR “doctor” OR “doctors” OR “nurse” OR “nurses” OR “medical” OR “medical staff” OR “medical assistant” OR “health personnel” OR “health care personnel” OR “healthcare personnel” OR “health care worker” OR “health care workers” OR “healthcare worker” OR “healthcare workers” OR “health worker” OR “health workers” OR “healthcare professionals” OR “medical care personnel” AND “knowledge” AND “awareness” (Supplementary Table 1) for the MEDLINE search. We also scanned through cross-references of identified primary studies and review articles for eligible studies.

### Eligibility and Exclusion Criteria

We included studies according to the following criteria; Population/Intervention/Comparator/Outcome/Study Design (PICOS) approach. For this review, we included HCWs (P) defined as individuals such as doctors, dentists, nurses, midwives, medical personnel, medical assistant or laboratory scientists who are in direct contact with the following: (i) patient bodily fluids or biological samples such as blood, saliva, sperm, sweat and stool, (ii) new-born delivery process in which mother-to-child transmission was possible via a transplacental route, and (iii) people who were exposed sexually, and (iv) sharp or needle-stick injuries. Instead of an intervention (I), we included observational studies that report the seroprevalence of HBV with either one or both of the components of knowledge or awareness in relation to HBV seroprevalence. There was no comparator (C), and lastly, the determined outcome (O) was seroprevalence of hepatitis B infection. We considered all articles published in English language. When numerous studies with the same cohort were conducted, the research with the most detailed information on the participants or the largest number of participants was chosen.

We excluded abstracts, letters to the editor, reviews, commentaries, editorials, and studies without either primary data or described study methods. We excluded systematic reviews, non-empirical studies, conference, abstracts, editorials, commentaries, book reviews, and abstracts not accompanied by a full text.

### Study Selection

Two review authors (NHM and NAM) independently screened all titles and abstracts to look for potential studies found through the search and coded them as “retrieve” (eligible or potentially eligible/unclear) or “do not retrieve”. Two more review authors (NSMD and MHAM) independently retrieved full-text study reports/publications to identify studies for inclusion, and to identify and record reasons for studies' exclusion. When there were disagreements, a consensus was obtained through discussion with a third reviewer (MRAH).

### Data Extraction Process and Data Items

Two review authors (FNL and TA) independently evaluated each included study ‘s methodological quality and extracted data using a piloted form; discrepancies were resolved through discussion with a third review author (NML). Data on the year of publication, country of origin, study design, sample size, sampling procedure (if available), study period, and setting (country/continent/region) were extracted using a standardized data collection form.

### Risk of Bias in Included Studies

To assess the risk of bias for each study, a domain-based questionnaire developed from the Newcastle-Ottawa Scale (NOS) was used to assess methodological quality (26) (Supplementary Table 2). In the following domains: participant selection (selection bias), sample size justification (selection bias), outcome measurement (detection bias), and confounding adjustment, we evaluated the risk of bias as low, moderate, high, or uncertain. We assigned a score of 7 and above as good quality, and below 6 as having some concerns to determine the overall quality.

### Data Synthesis and Analysis

We used Stata software version 16 for all statistical analysis (27). The pooled prevalence rates, as well as their 95% confidence intervals, were calculated using a random-effects model (28). The heterogeneity of the studies was assessed using I^2^ statistics and Cochran's Q Test (29). The I^2^ statistics were used to assess the explained variance attributable to study heterogeneity, with I^2^ score of 25.0, 50.0 and 75.0% denoting low, moderate and high, respectively (28, 30).

## Results

### Identification of Studies

**Figure 1:**
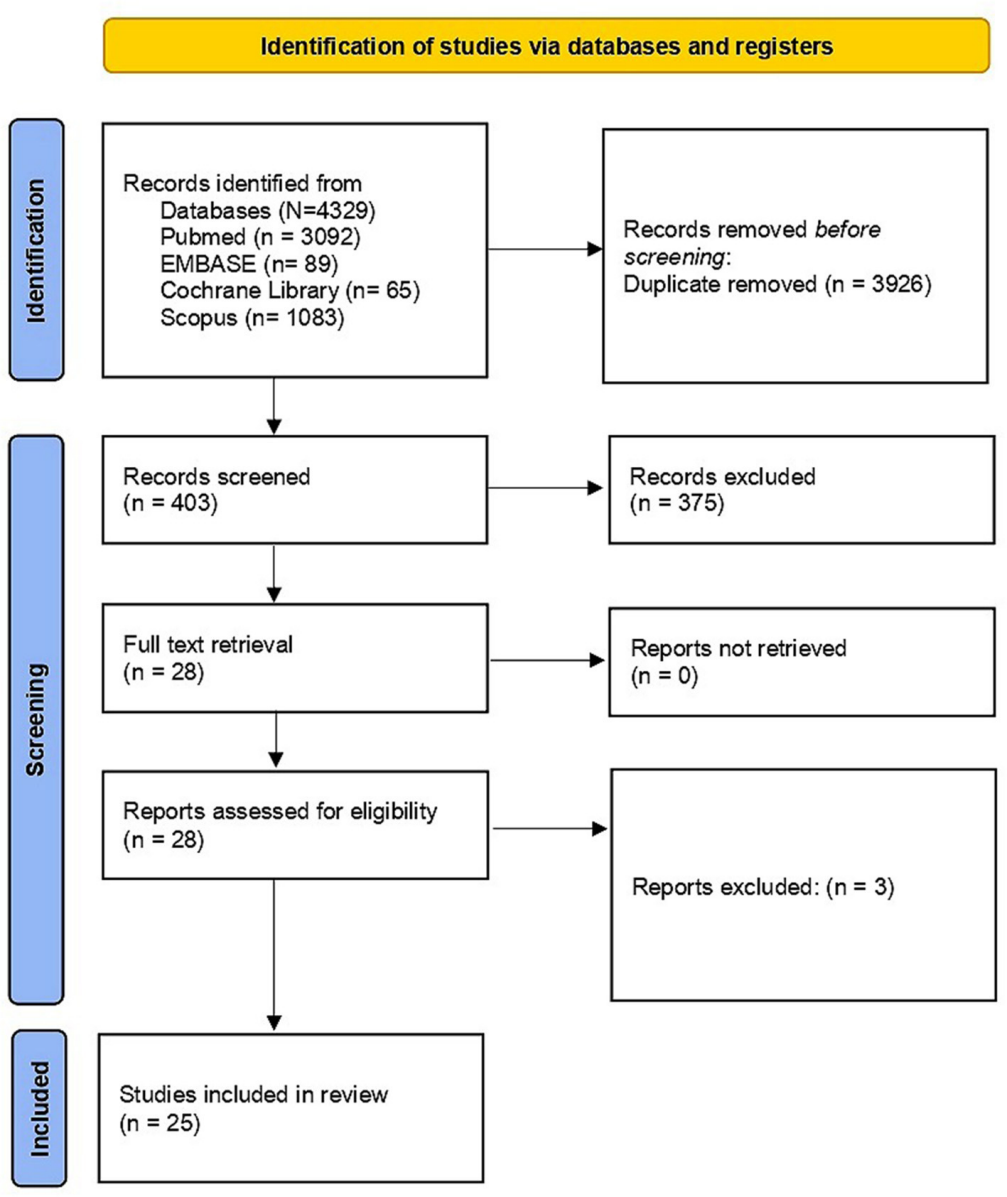
PRISMA flow diagram on selection process. From: Page et al. (24). For more information, visit: http://www.prisma-statement.org/.

We identified a total of 4,329 studies published between January 1997 and September 2021. We could not find any studies that evaluated seroprevalence of Hepatitis B among HCW and its association with knowledge, awareness and attitude of HBV in the Americas, Europe, Eurasia, Australia, or Antarctica regions. We excluded duplicates and collated multiple reports of the same study so that each study, rather than individual report, is the focus of our analysis. We meticulously documented the selection process to complete a PRISMA flow diagram (Figure 1). We shortlisted 25 studies from a pool of 28 for an in-depth full-text examination of their suitability.

### Characteristics of Included Studies

Among the 25 included studies, nineteen were from Africa (10, 14, 20, 31–46) and six from Asia (47–52). The studies were conducted across eleven countries with a total of 10,043 participants. The characteristics of the included studies are depicted in Table 1.

**Table 1:** List of included studies.

**No**	**References**	**Study population (country)**	**Continent/region**	**Sample size**	**Seroprevalence (%) /HBV marker**	**Knowledge/ Awareness findings**
1	Djeriri et al. (31)	Morocco	Africa	276	1.0 (HBsAg)	Awareness: Overall good awareness. 95% aware the complications of chronic Hepatitis B, 68% aware Hepatitis could be fatal, 100% aware HBV can be transmitted by blood transfusion, 85% aware HBV transmitted by sexually transmitted disease and 97% by vertical transmission. 93% aware condom as an effective for prevention, 87% aware washing hands prevent HBV transmission and 96% aware the use of disposal gloves to prevent Hepatitis B
2	Shao et al. (32)	Tanzania	Africa	442	5.7 (HBsAg)	Knowledge: Overall average knowledge. Quarter (25.4%) had good knowledge and about half (49.6%) had fair knowledge about HBV infection. Most of the participants (85.9%) correctly identified that HBV is more contagious than HIV, while (91.3%) knew that there is effective and safe hepatitis B vaccine. Knowledge questions: HBV transmit via sexual intercourse and partner, known as blood-borne pathogen via accidental exposure to blood and its product, needle stick, broken skin, mucous membrane, infected blood, oral-fecal route, mom to fetus, immunoglobulin, and vaccine. Awareness: Overall poor awareness. 17.9% of participants were aware, unprotected sex with multiple partners was the risks for HBV infection.
3	Hebo et al. (33)	Ethiopia	Africa	240	4.4 (HBsAg)	Knowledge: Overall good knowledge on the virus (73.9%) including the transmission and the treatment. 26.1% had average knowledge
4	Desalegn and Selsassie (34)	Ethiopia	Africa	254	2.4 (HBsAg)	Knowledge: Overall good knowledge of universal precautions (UPs). 52.4% consistent use of gloves was reported by of the respondents, 61.0% concerning needle stick injury (NSI) and from other sharp injury and 50.0% had a history of NSI and sharp injury. 80.7% and 42.5% of HCWs knew about universal precaution guideline and were trained on infection prevention, respectively.
5	Anagaw et al. (35)	Ethiopia	Africa	100	6.0 (HBsAg)	Awareness: Overall good awareness. Aware on the viral hepatitis transmission via sexual contact, sharing special tools (i.e., eye goggle, thick gloves, protective gown, tooth brushes, shaving razor, etc.) and intravenous drug abuse.
6	Abiola et al. (10)	Nigeria	Africa	134	1.5 (HBsAg)	Knowledge: Overall good knowledge (56.7%) and 43.3% with average knowledge
7	Ngekeng et al. (36)	Nigeria	Africa	188	5.0 (HBsAg)	Knowledge: Overall average knowledge. 58.72% had good knowledge and 41.28% had poor knowledge. Average knowledge on the HBV transmission (66.9%) and good knowledge (80.0%) know HBV cause liver disease
8	Osagiede et al. (20)	Nigeria	Africa	280	1.4 (HBsAg)	Knowledge: Overall average knowledge. 32.5% had poor, 20% had average and 47.5% have good knowledge. Awareness: Overall good awareness with 86.4% aware about HBV.
9	Ijoma et al. (37)	Nigeria	Africa	3,123	2.3 (HBsAg)	Knowledge: Overall good knowledge on HBV infection (97.0%) and 68.1% correctly identify risk factors and transmission. Poor knowledge on sexual intercourse and sharp objects
10	Ogundele et al. (14)	Nigeria	Africa	209	6.7 (HBsAg)	Knowledge: Overall adequate knowledge with 61.7% had adequate knowledge while 38.3% had poor knowledge range. The knowledge score was only significantly associated with work duration (*p* = 0.018). 89% of the participants ever heard of HBV prior to the study. Awareness: Overall good awareness with 83.7% were aware that HBV is contagious, only 125(59.8%) described it as a lethal disease
11	Oladokun et al. (38)	Nigeria	Africa	140	5 (HBsAg)	Knowledge: Overall good knowledge of the infection though some have had needle stick injury (12.14%) Awareness: Overall good awareness. Aware of the infection (92.86%) and its modes of transmission (72.86%).
12	Muhammad et al. (39)	Nigeria	Africa	283	6.0 (HBsAg)	Knowledge: Overall adequate knowledge with 58.3% had knowledge on HBV and 41.7% with poor knowledge Awareness: Overall good awareness with high awareness level observed in individuals wearing hand gloves. However, 70.1% do not recap needles after the injection
13	Amiwero et al. (40)	Nigeria	Africa	248	1.3 (HBsAg)	Awareness: Overall good awareness with 70.6% aware of various types of hepatitis and suggested that awareness increased with the increased of education level.
14	Mbaawuaga et al. (41)	Nigeria	Africa	255	10.6 (HBsAg)	Awareness: Overall good awareness with 79.6% had awareness about HBV infection.
15	Akazong et al. (42)	Cameroon	Africa	395	10.6 (HBsAg)	Knowledge: Overall average knowledge. 32.4% had poor knowledge while 67.6% had average knowledge
16	Rodrigue et al. (43)	Cameroon	Africa	171	7.0 (HBsAg)	Knowledge: Overall, good knowledge. 94.7% had good knowledge and 5.3% had poor knowledge. Good knowledge with 93% know it's come from virus. Most of HCWs believed HBV cause by sexual intercourse (96.5%), scarifications (34.5%) and blood exposure (19.3%)
17	Tatsilong et al. (44)	Cameroon	Africa	100	11.0 (HBsAg)	Knowledge: Overall had average knowledge. 47% had good knowledge in HBV mostly in men (3.2 times than women). Higher education, knowledge on the present of HB vaccine, needle injury, knowing the mode of HBV transmitted are named as a contribution factor to higher knowledge of HBV.
18	Qin et al. (45)	Sierra Leone	Africa	211	10.0 (HBsAg)	Knowledge: Overall average knowledge with 29.0% had poor knowledge on transmission, preventive HBV measure (44.1%). Longer working experience is associated with greater knowledge & medical doctor. Awareness: Overall poor awareness with 77.3% not aware about HBV clinical outcome.
19	Massaquoi et al. (46)	Sierra Leone	Africa	446	8.7 (HBsAg)	Knowledge: Overall good knowledge with 90.4% of participants were aware that hepatitis B could cause liver cancer. About 96.9% healthcare workers were concerned about their risk of hepatitis B at work
20	Mangkara et al. (47)	Laos	Asia	317	5.0 (HBsAg)	Knowledge: Overall poor knowledge with 20% of dentists and 45% of assistants were unaware that HBV can be transmitted by blood. 8.2% of the dentists and 18.1% of assistants were not familiar or did not recognize serology as a way to test for Hepatitis B infection.
21	Nguyen et al. (48)	Vietnam	Asia	203	9.8 (HBsAg)	Knowledge: Overall good knowledge on the mode of HBV transmission. Majority believed that asymptomatic people can have chronic HBV or HCV infection (89%) and that HBV-HCV are lifelong infections which can cause liver cancer (95%) and can be lethal (86%). Physicians exhibit better knowledge than nurses or midwives and other HCWs.
22	Ptil et al. (49)	India	Asia	555	0.2 (HBsAg)	Awareness: Overall good awareness, with 98% aware of health consequences of HBV accidental exposure (needle prick and post exposure prophylaxis) and concerned about follow up
23	Aziz et al. (52)	India	Asia	250	2.4 (HBsAg)	Knowledge: Overall good knowledge with 90% know HBV can be transmission in hospital, needle stick (62%), sexual (59%), vertical (71%), hand washing (13%), precaution to avoid needle stick injury (23%), wear gloves (30%), proper vaccine (14%), regular screen (10%), no knowledge (1%). About quarter of them had needle stick injury during hospital job but few were tested against it. Less than half of them were previously vaccinated for HBV but majority of them knew about the risk of transmission of HBV, HCV and I-IIV during hospital job.
24	Memon et al. (51)	Pakistan	Asia	923	4.7 (HBsAg)	Knowledge: Overall poor knowledge regarding the importance of HBV prevention, 20%
25	Alqahtani et al. (50)	Saudi Arabia	Asia	300	8.7 (HBsAg)	Knowledge: Overall average knowledge observed among HCWs regarding occupationally transmitted blood-borne diseases, safe injection practices, and standard precautions to prevent occupationally transmitted blood-borne infections. Awareness: Overall good awareness with 99.0% of HCWs were aware of all blood-borne diseases, 53.0% felt all safe injection practices that may protect them and 72.6% said all standard isolation precautions to prevent occupationally transmitted blood-borne infections.

### Quality Assessment of Included Studies

Two reviewers (FNL and MHAM) independently assessed the quality of the included studies using an adapted version of the NOS for prevalence studies. The quality of evidence was rated as low to begin with, due to the non-randomized nature of the study. The quality of evidence in the outcomes were based on the NOS criteria, where a maximum score of 4 stars for selection, 2 stars for comparability, and 3 stars for exposure and outcome assessment. Studies with fewer than 2 stars were considered low quality; 2 to 6 stars, moderate quality; and 7 stars and more, high quality. Twenty-one studies were observed to be of good quality (NOS score 7 and above), and four studies were identified as fair quality (NOS score between 2 and 6 inclusive) (Supplementary Table 2).

### The Estimates of Pool Seroprevalence of Hepatitis B

**Figure 2:**
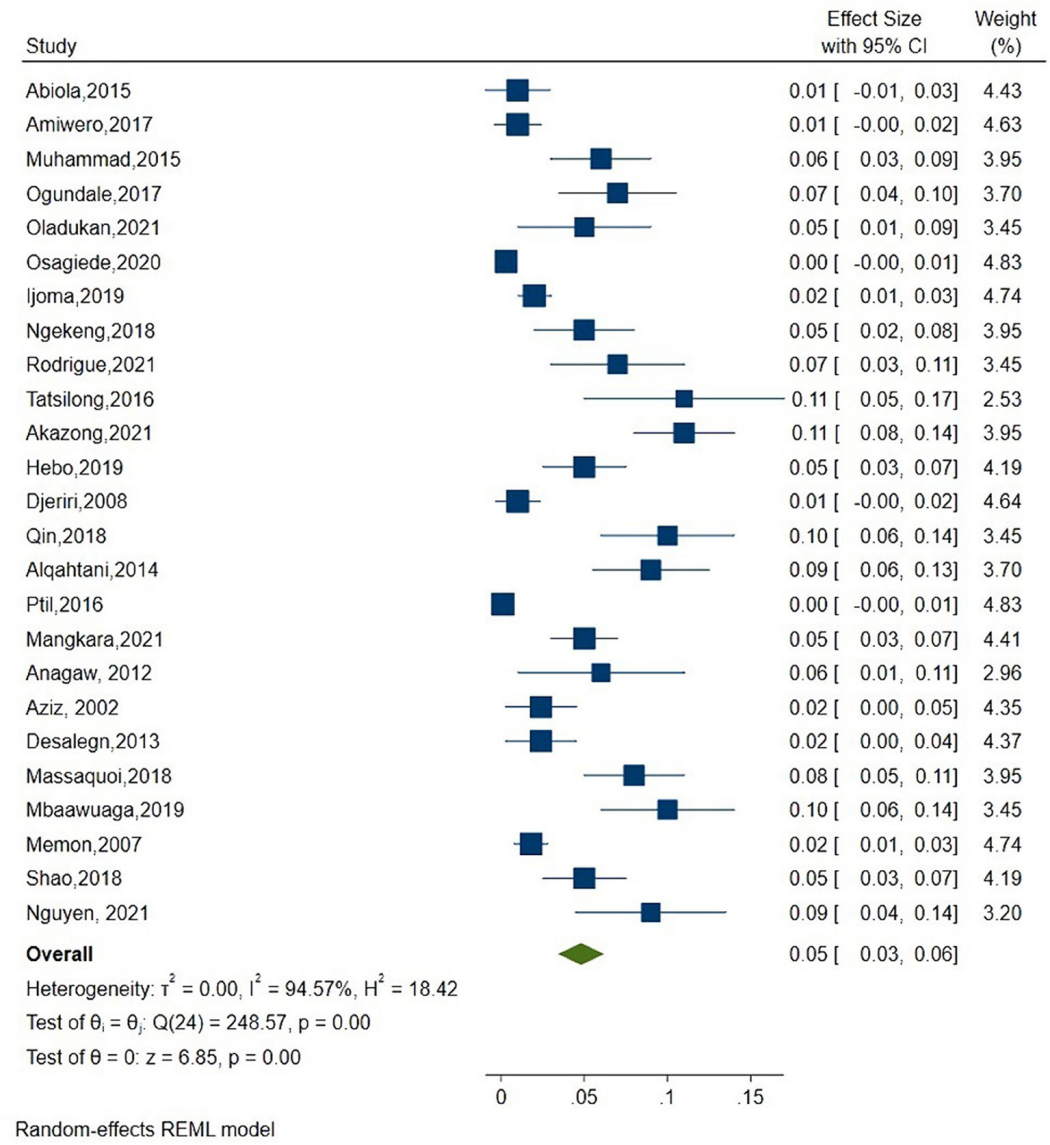
Forest plot of overall seroprevalence estimate of the Hepatitis B infection among healthcare workers.

**Figure 3:**
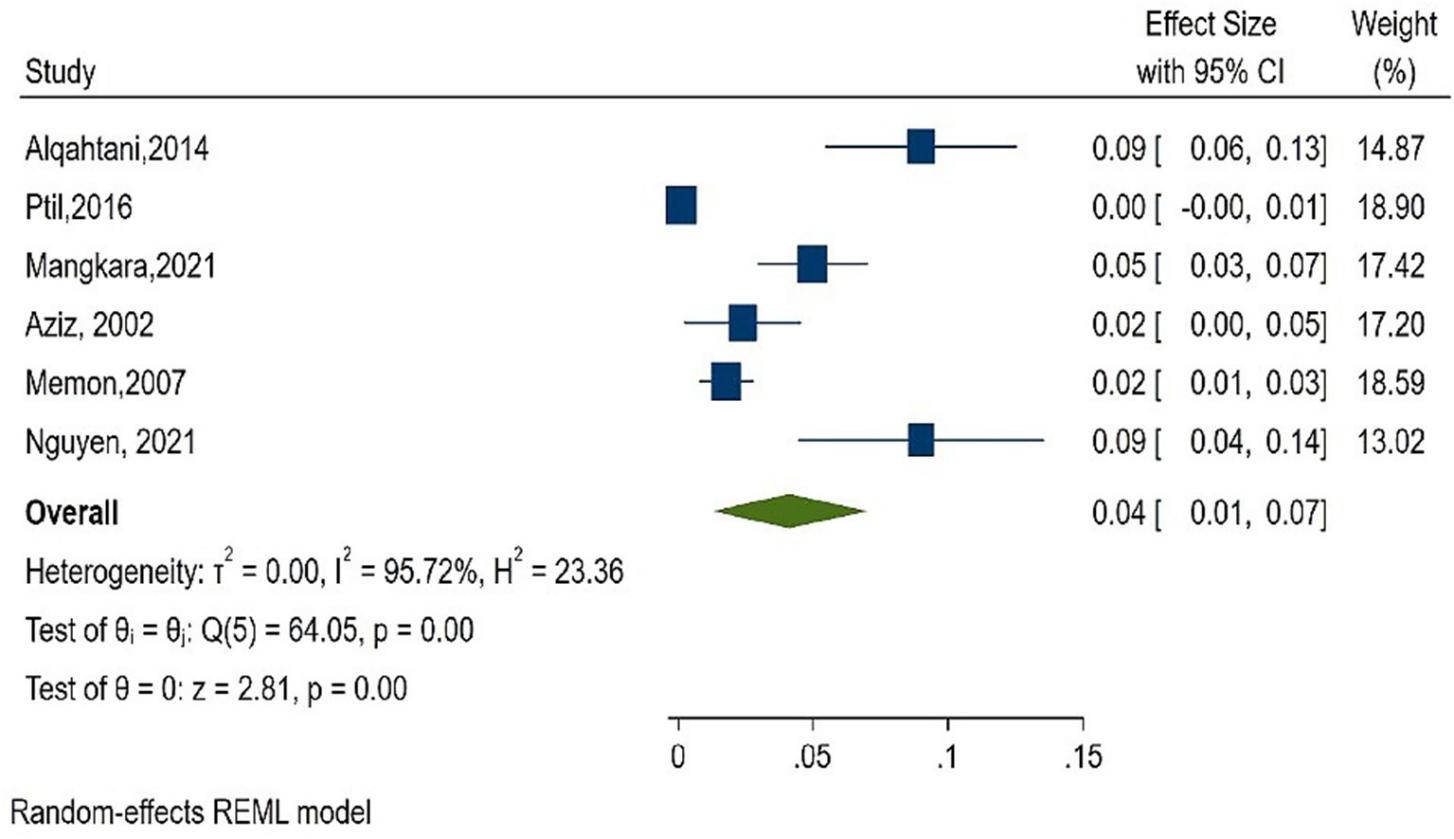
Forest plot of seroprevalence estimate of the Hepatitis B infection among healthcare workers in Asia.

**Figure 4:**
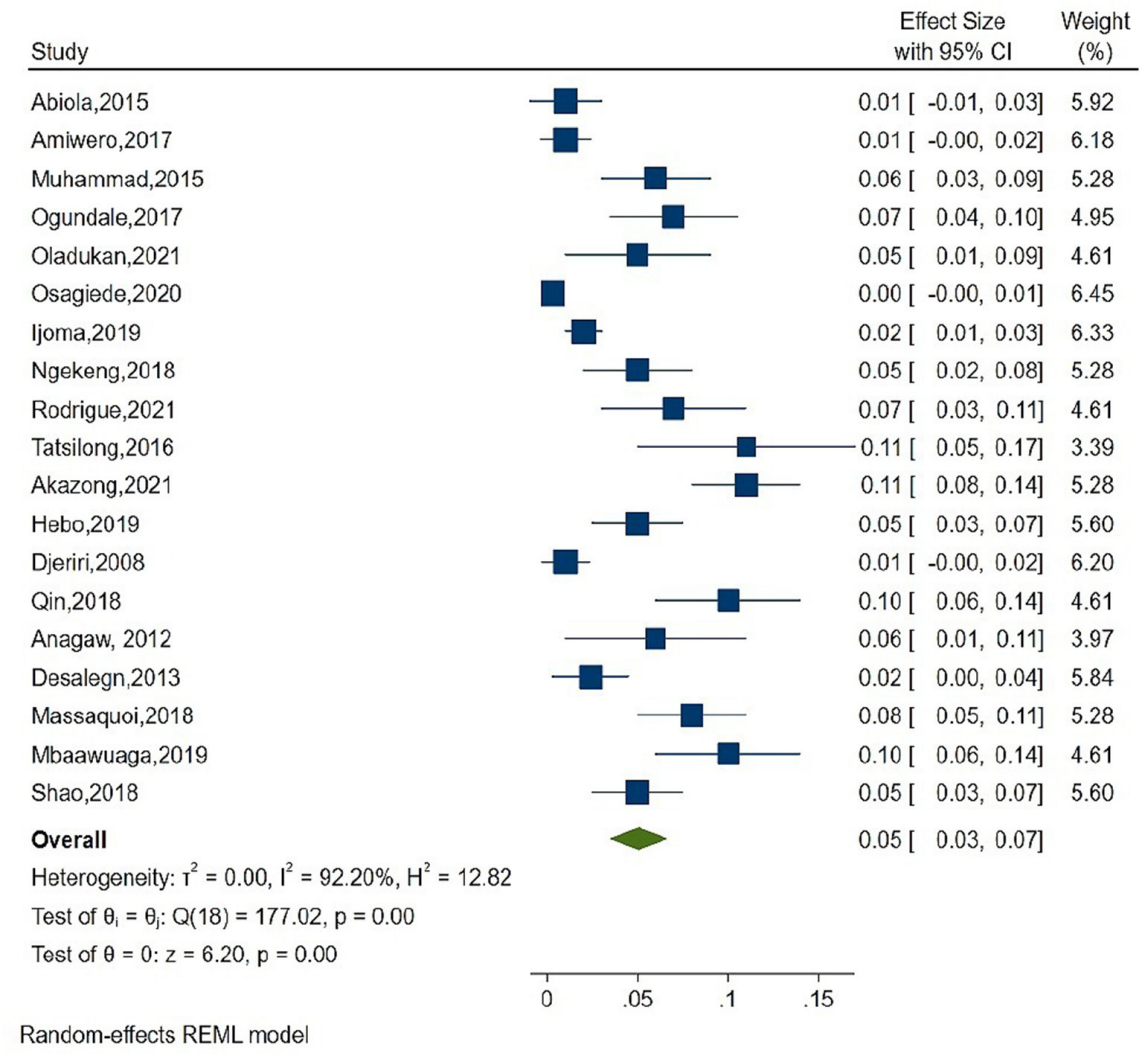
Forest plot of seroprevalence estimate of the Hepatitis B infection among healthcare in Africa region.

As shown in Figure 2, the overall estimate for pooled seroprevalence of Hepatitis B was 5.0% (95% CI: 0.03–0.06), with a high-level of heterogeneity between studies (I^2^ = 94.6%, *P* = 0.001). The study was divided into two parts: Africa and Asia. In Asia, the overall pooled seroprevalence of Hepatitis B was 4.0% (95% CI: 0.01–0.07) with a high-level of heterogeneity between studies (I^2^ = 95.7%, *P* = 0.001) (Figure 3), whereas in Africa, the overall pooled seroprevalence of Hepatitis B in Africa was 5.0% (95% CI: 0.03–0.07) with a high-level of heterogeneity between studies (I^2^ = 92.2%, *P* = 0.001) (Figure 4). The subgroup analysis was performed in Africa since there were nineteen publications categorized under the African region. We pooled the seroprevalence of Hepatitis B in African countries with two or more publications for this study. This subgroup analysis was performed in Nigeria, Ethiopia, and Cameroon (Supplementary Figures 1–3). In Nigeria, the overall pooled seroprevalence of hepatitis B was 4.0% (95% CI: 0.01–0.06) with high-level heterogeneity (I^2^ = 94.4%, *P* = 0.001) (Supplementary Figure 1). Ethiopia had a pooled seroprevalence of 4.0% (95% CI: 0.02–0.06), but the level of heterogeneity was moderate (I^2^ = 42.6%, *P* = 0.19) (Supplementary Figure 2). Cameroon had the highest overall pooled seroprevalence of Hepatitis B in Africa, at 8.0% (95% CI: 0.05–0.11), with a moderate level of heterogeneity between studies (I^2^ = 63.7%, *P* = 0.03) (Supplementary Figure 3).

### Knowledge and Awareness on Hepatitis B Infection Among Healthcare Workers

There were twenty studies that reported findings on seroprevalence and knowledge, with fifteen from Africa and five from Asia. Some of the studies mentioned the knowledge level (good, average, or poor knowledge) in the publication. In Africa, the majority of the studies found that participants have a strong knowledge of hepatitis B infection, with eight studies (53.3%) reporting good knowledge and seven studies (46.7%) reporting average knowledge. In Asia, five studies reported seroprevalence and knowledge findings, with two studies (40.0%) reporting good knowledge, one study (20.0%) reporting average knowledge, and two studies (40.0%) reporting poor knowledge. Seven studies in Africa found that the average knowledge and seroprevalence were 1.4 and 11.0%, respectively. Surprisingly, few African studies demonstrated good knowledge despite the fact that their HBV infection rate was higher than 6.7% (14, 42, 45). The average knowledge was reported in the cohort with a seroprevalence of 8.7% in the Asia region (49) Memon et al. (51) and Mangkara et al. (47) found poor knowledge in their participants with 4.7% and 5.0% HBV seroprevalence, respectively. However, Nguyen et al. (48), who found the highest seroprevalence (9.8%), claimed that participants had a good knowledge of HBV infection (48). The assessment of knowledge among HCWs were presented in the Supplementary Table 3.

There were eleven studies that found an association between seroprevalence and participants' level of awareness, including nine (81.8%) from Africa and two (18.2%) from Asia. Some of the studies mentioned the participants' level of awareness (good, average, or poor awareness). The scale for awareness level was adopted from Vaishali et al. (53) for those studies that did not mention it. Nine studies (81.8%) were reported to have good awareness, whereas two (18.2%) in the Africa region were found to have poor awareness. Seroprevalence was poor in Tanzania (32) and Sierra Leone (45), with 5.7 and 10.0%, respectively. Even though three publications had higher seroprevalence than Shao et al. (32) and Qin et al. (45), the publications showed good awareness: 6.0% Anagaw et al. (35), 6.7% Ogundele et al. (14), and 10.6% Mbaawuaga et al. (41). Supplementary Table 4 summarizes the awareness evaluation, the instrument employed, and the conclusion.

## Discussion

Healthcare workers have a higher risk of contracting HBV infection than the general population. Simultaneously, they play a vital role in preventing and controlling HBV infection by disseminating and transmitting HBV knowledge to the public, as well as assisting in behavior changes that may aid in infectious diseases prevention (54). After utilizing NOS to assess methodological quality, only 21 out of the 25 studies included in this review showed a good risk of bias, while another four studies exhibited a fair risk of bias. Healthcare workers must consequently have a goof level of knowledge and awareness of HBV to limit their own and the public's risk of infection. According to Rayate et al. (55), the majority of HCWs are unaware that the virus can survive outside the body for seven days (55). The same study also reported that only 27.78% of HCWs are aware that the virus can survive in dried blood type form (55). Several factors influence the likelihood of getting hepatitis B, including the prevalence of the virus in the environment or in people's behavior, the frequency of blood and body fluid exposure to HCWs, HBV infectivity (14), geographical location, and host factors (10).

In comparison to other regions, the current study found that African countries have a high seroprevalence of HBV infection (Figures 3, 4). Medical doctors, dentists, nurses, and laboratory workers made up the majority of HCWs infected with HBV. There were also cases of HBV infection among technicians, nurse assistant, cleaning operators, and housekeeping staff (17, 41, 48, 49). Accidental exposure to blood and blood products, occupational injuries such as needle-sticks and other injuries from sharp objects, lack of experience or practice with HBV infection, and not having been vaccinated were all risk factors for high seroprevalence of HBV infection in certain places (32–34, 36, 47, 51). Mahamat et al. (21) published a study on global seroprevalence among HCWs that was similar to ours. Our study, on the other hand, shows a link between HBV seroprevalence and HCWs knowledge or awareness of HBV infection, which was not addressed in the previous study (21).

Despite the high seroprevalence observed in a few studies, other publications claimed good knowledge or awareness of HBV infection (14, 35, 41, 43, 46). High HBV seroprevalence has been attributed to a lack of knowledge about HBV transmission routes in one study in Cameroon (42). Surprisingly, despite the lower seroprevalence, some studies reported average or poor knowledge or awareness of HBV infection (32, 39). This finding showed an inconsistency between the level of seroprevalence and the level of knowledge or awareness among HCWs. Notwithstanding the inconsistency, it is critical to increase HBV knowledge and awareness among HCWs (50).

A few approaches to increase an awareness of HBV infection among HCWs include strengthening immunization program, regularly screen the HBsAb and HBsAg of HCWs, media involvement, continuous medical educations, and provide trainings to the HCWs (14, 32, 33, 46, 50). This will encourage safer work practices and a higher degree of compliance with hospital policies. By assessing the level of knowledge among HCWs, not only is the general public indirectly examined, but also preventative implementation is improved. Separately, the long years of hospital service have contributed to raising increasing hepatitis B infection awareness. Furthermore, HCWs' lack of knowledge regarding Hepatitis B could have a significant impact on safety behaviors, such as vaccination. As a result, HCW awareness of Hepatitis B is vital, as knowledge plays a key role in changing prevention-related behavior (22). Lack of training or seminars for HCWs, insufficient information, or a poor awareness of HBV infection could all contribute to the lack of knowledge among HCWs. Moreover, insufficient health education programs and obtaining unreliable Hepatitis B information from friends, relatives, and co-workers may increase the likelihood of acquiring incorrect information (42). To increase HBV knowledge among HCWs, improvement in clinical practice, 53 training, and practical skills are required (56, 57).

The present study had limitations. First, we were unable to locate reports on the HBV seroprevalence and levels of knowledge or awareness in the developing countries. Thus, we were unable to compare the findings worldwide. Second, in some research, differences in score and inability to score on the level of knowledge or awareness may result in inconsistent conclusions that are either good, average, or poor. As a result, we were unable to determine some research' scores and compare them to seroprevalence.

In conclusion, hepatitis B virus was shown to be present in 4.0–5.0 % of the population tested, with an apparent higher prevalence in African countries than in Asian countries. Some HCWs were still infected with HBV despite having strong knowledge and awareness of HBV infection. Improved epidemiological data collection can help determine and identify key risk factors for a more effective public health response. Thus, if enough people are exposed to Hepatitis B virus knowledge, awareness, attitude, and practice, the goal of eliminating viral hepatitis by 2030 may be achieved.

## Data Availability Statement

The original contributions presented in the study are included in the article/Supplementary Material, further inquiries can be directed to the corresponding author/s.

## Author Contributions

NHM and NAM carried out the study design, study selection, data extraction, and statistical analysis and drafted the manuscript. NSMD and MHAM participated in the study selection and data extraction and drafted the manuscript. FNL and TA evaluated the quality of included studies. MRAH and NML participated in the discussion for any discrepancies and supervised the study. All authors read and approved the final manuscript.

## Funding

The open-access publication fee was supported by the National Institutes of Health, Ministry of Health, Malaysia.

## Conflict of Interest

The authors declare that the research was conducted in the absence of any commercial or financial relationships that could be construed as a potential conflict of interest.

## Publisher's Note

All claims expressed in this article are solely those of the authors and do not necessarily represent those of their affiliated organizations, or those of the publisher, the editors and the reviewers. Any product that may be evaluated in this article, or claim that may be made by its manufacturer, is not guaranteed or endorsed by the publisher.
