# Supplementary material for: Seroprevalence of Hepatitis B Among Healthcare Workers in Asia and Africa and Its Association With Their Knowledge and Awareness: A Systematic Review and Meta-Analysis
Source: Front Public Health. 2022 Apr 28;10:859350. doi: 10.3389/fpubh.2022.859350 (PMC9096243; doi:10.3389/fpubh.2022.859350)
Supplement: Supplementary file 1 [file Data_Sheet_1.PDF]

Supplementary Table 1: Key search strategy

**Key search strategy**	**Search date**
The databases searched include PubMed, Google Scholar, ProQuest and Cochrane library<br><br>**Keywords for PubMed:**<br>‘‘hepatitis B’’ OR ‘‘HBV’’ OR ‘‘hepatitis B virus’’ AND ‘‘etiology’’ OR “aetiology” OR ‘‘prevalence’’ OR ‘‘epidemiology’’ OR “infection” AND ‘‘healthcare workers’’ OR ‘‘healthcare’’ OR “doctor” OR “doctors” OR “nurse” OR “nurses” OR “medical” OR “medical staff” OR “medical assistant” AND “knowledge” AND “awareness”<br><br>**Keywords for Google Scholar:**<br>‘‘hepatitis B’’ OR ‘‘HBV’’ OR ‘‘hepatitis B virus’’ AND ‘‘etiology’’ OR “aetiology” OR ‘‘prevalence’’ OR ‘‘epidemiology’’ OR “infection” AND ‘‘healthcare workers’’ OR ‘‘healthcare’’ OR “doctor” OR “doctors” OR “nurse” OR “nurses” OR “medical” OR “medical staff” OR “medical assistant” AND “knowledge” AND “awareness”<br><br>**Keywords for ProQuest:**<br>‘‘hepatitis B’’ OR ‘‘HBV’’ OR ‘‘hepatitis B virus’’ AND ‘‘etiology’’ OR “aetiology” OR ‘‘prevalence’’ OR ‘‘epidemiology’’ OR “infection” AND ‘‘healthcare workers’’ OR ‘‘healthcare’’ OR “doctor” OR “doctors” OR “nurse” OR “nurses” OR “medical” OR “medical staff” OR “medical assistant” AND “knowledge” AND “awareness”	August to October 2021 (article searching was performed during this six-month period)

**Keywords for Cochrane library:**<br><br>‘‘hepatitis B’’ OR ‘‘HBV’’ OR ‘‘hepatitis B virus’’ AND ‘‘etiology’’ OR “aetiology” OR ‘‘prevalence’’ OR ‘‘epidemiology’’ OR “infection” AND ‘‘healthcare workers’’ OR ‘‘healthcare’’ OR “doctor” OR “doctors” OR “nurse” OR “nurses” OR “medical” OR “medical staff” OR “medical assistant” AND “knowledge” AND “awareness”	

PICOS

**P**	Participant	Healthcare workers (HCWs); doctors, dentists, nurses and medical assistants
**I**	Intervention	Any component of prevalence and knowledge or awareness
**C**	Comparator	Any component without prevalence and knowledge or awareness
**O**	Outcome	Primary:<br>Incidence or prevalence of typhoid disease<br>Secondary:<br>Level of knowledge or awareness of HCWs
**S**	Study	Any observational studies except commentaries, editorial, case series and systematic review
