# Supplementary material for: Seroprevalence of Hepatitis B Among Healthcare Workers in Asia and Africa and Its Association With Their Knowledge and Awareness: A Systematic Review and Meta-Analysis
Source: Front Public Health. 2022 Apr 28;10:859350. doi: 10.3389/fpubh.2022.859350 (PMC9096243; doi:10.3389/fpubh.2022.859350)
Supplement: Supplementary file 2 [file Data_Sheet_2.PDF]

Supplementary Table 2: Newcastle-Ottawa Quality Assessment Form for Cohort Studies

No	Author/year	Selection				Comparability	Outcome			Quality score
		S1	S2	S3	S4		O1	O2	O3	
1	Djeriri et al. (2008)	*	*	*	*	*		*	*	Good
2	Shao et al. (2018)	*	*	*	*	*		*	*	Good
3	Hebo et al. (2019)	*	*	*	*	*		*	*	Good
4	Desalegn et al (2013)	*	*	*	*	*		*	*	Good
5	Anagaw et al (2012)	*	*	*	*	*		*	*	Good
6	Abiola et al. (2015)	*	*	*	*	*		*	*	Good
7	Ngekeng et al. (2018)	*	*	*	*	*		*	*	Good
8	Osagiede et al. (2020)	*	*	*	*	*		*	*	Good
9	Ijoma et al. (2019)	*	*	*	*	*		*	*	Good
10	Ogundele et al. (2017)	*	*	*	*	*		*	*	Good
11	Oladokun et al. (2021)	*	*	*	*	*		*	*	Good
12	Muhammad et al. (2015)	*	*	*	*			*	*	Fair
13	Amiwero et al. (2017)	*	*	*	*	*		*	*	Good
14	Mbaawuaga et al. (2019)	*	*	*	*	*		*	*	Good
15	Akazong et al. (2021)	*	*	*	*	*		*	*	Good
16	Rodrigue et al. (2021)	*	*	*	*	*		*	*	Good
17	Tatsilong et al. (2016)	*	*	*	*	*		*	*	Good
18	Qin et al. (2018)	*	*	*	*	*		*	*	Good
19	Massaquoi et al. (2018)	*	*	*	*	*		*	*	Good
20	Mangkara et al. (2021)	*	*	*	*	*		*	*	Good
21	Nguyen et al. (2021)	*	*	*	*	*		*	*	Good
22	Ptil et al. (2016)	*	*		*			*	*	Fair
23	Aziz et al. (2002)	*	*		*			*	*	Fair
24	Memon et al. (2007)	*	*	*	*	*		*	*	Good
25	Alqahtani et al. (2014)	*	*	*	*			*	*	Fair

Newcastle-Ottawa Quality Assessment Form for Cohort Studies

Note: A study can be given a maximum of one star for each numbered item within the Selection and Outcome categories. Amaximum of two stars can be given for Comparability.

Selection

1) Representativeness of the exposed cohort
   - *a)* Truly representative ***(one star)***
   - *b)* Somewhat representative ***(one star)***
   - *c)* Selected group
   - *d)* No description of the derivation of the cohort

2) Selection of the non-exposed cohort
   - *a)* Drawn from the same community as the exposed cohort ***(one star)***
   - *b)* Drawn from a different source
   - *c)* No description of the derivation of the non exposed cohort

3) Ascertainment of exposure
   - *a)* Secure record (e.g., surgical record) ***(one star)***
   - *b)* Structured interview ***(one star)***
   - *c)* Written self report
   - *d)* No description
   - *e)* Other

4) Demonstration that outcome of interest was not present at start of study
   - *a)* Yes ***(one star)***
   - *b)* No

Comparability

1) Comparability of cohorts on the basis of the design or analysis controlled for confounders
   - *a)* The study controls for age, sex and marital status ***(one star)***
   - *b)* Study controls for other factors (list)___________________________***(one star)***
   - *c)* Cohorts are not comparable on the basis of the design or analysis controlled for confounders

Outcome

1) Assessment of outcome
   - *a)* Independent blind assessment ***(one star)***
   - *b)* Record linkage ***(one star)***
   - *c)* Self report
   - *d)* No description
   - *e)* Other

2) Was follow-up long enough for outcomes to occur
   - *a)* Yes ***(one star)***
   - *b)* No
   - c)

Indicate the median duration of follow-up and a brief rationale for the assessment above:__________

3) Adequacy of follow-up of cohorts

a) Complete follow up- all subject accounted for ***(one star)***
b) Subjects lost to follow up unlikely to introduce bias- number lost less than or equal to 20% or description of those lostsuggested no different from those followed. ***(one star)***
c) Follow up rate less than 80% and no description of those lost
d) No statement

Thresholds for converting the Newcastle-Ottawa scales to AHRQ standards (good, fair, and poor):

Good quality: 3 or 4 stars in selection domain AND 1 or 2 stars in comparability domain AND 2 or 3 stars in outcome/exposure domain

Fair quality: 2 stars in selection domain AND 1 or 2 stars in comparability domain AND 2 or 3 stars in outcome/exposure domain

Poor quality: 0 or 1 star in selection domain OR 0 stars in comparability domain OR 0 or 1 stars in outcome/exposure domain

Checklist of information that should be included in new reports of global health estimates

Item #	Checklist item	Reported on page #
Objectives and funding		
1	Define the indicator(s), populations (including age, sex, and geographic entities), and time period(s) for which estimates were made.	6
2	List the funding sources for the work.	12
Data Inputs		
For all data inputs from multiple sources that are synthesized as part of the study:		
3	Describe how the data were identified and how the data were accessed.	6
4	Specify the inclusion and exclusion criteria. Identify all ad-hoc exclusions.	6
5	Provide information on all included data sources and their main characteristics. For each data source used, report reference information or contact name/institution, population represented, data collection method, year(s) of data collection, sex and age range, diagnostic criteria or measurement method, and sample size, as relevant.	Suppl. Table S1
6	Identify and describe any categories of input data that have potentially important biases (e.g., based on characteristics listed in item 5).	Suppl. Appendix 1
For data inputs that contribute to the analysis but were not synthesized as part of the study:		
7	Describe and give sources for any other data inputs.	-
For all data inputs:		
8	Provide all data inputs in a file format from which data can be efficiently extracted (e.g., a spreadsheet rather than a PDF), including all relevant meta-data listed in item 5. For any data inputs that cannot be shared because of ethical or legal reasons, such as third-party ownership, provide a contact name or the name of the institution that retains the right to the data.	7 – 11
Data analysis		
9	Provide a conceptual overview of the data analysis method. A diagram may be helpful.	23
10	Provide a detailed description of all steps of the analysis, including mathematical formulae. This description should cover, as relevant, data cleaning, data pre-processing, data adjustments and weighting of data sources, and mathematical or statistical model(s).	7 - 11
11	Describe how candidate models were evaluated and how the final model(s) were selected.	5 - 7
12	Provide the results of an evaluation of model performance, if done, as well as the results of any relevant sensitivity analysis.	7 - 11
13	Describe methods for calculating uncertainty of the estimates. State which sources of uncertainty were, and were not, accounted for in the uncertainty analysis.	-
14	State how analytic or statistical source code used to generate estimates can be accessed.	-
Results and Discussion		
15	Provide published estimates in a file format from which data can be efficiently extracted.	
16	Report a quantitative measure of the uncertainty of the estimates (e.g. uncertainty intervals).	8 - 11
17	Interpret results in light of existing evidence. If updating a previous set of estimates, describe the reasons for changes in estimates.	8 - 11
18	Discuss limitations of the estimates. Include a discussion of any modelling assumptions or data limitations that affect interpretation of the estimates.	12 - 14

This checklist should be used in conjunction with the GATHER statement and Explanation and Elaboration document, found on gather-statement.org
