# Supplementary material for: Seroprevalence of Hepatitis B Among Healthcare Workers in Asia and Africa and Its Association With Their Knowledge and Awareness: A Systematic Review and Meta-Analysis
Source: Front Public Health. 2022 Apr 28;10:859350. doi: 10.3389/fpubh.2022.859350 (PMC9096243; doi:10.3389/fpubh.2022.859350)
Supplement: Supplementary file 3 [file Data_Sheet_3.PDF]

Supplementary Table 3. The assessment of knowledge level among healthcare workers.

No	Author/Year	Knowledge assessment	Tool used	Score	Finding
1	Shao et al. (2018)	Self-administered questionnaire	Knowledge measurements adopting an approach used by Abdul Hakeem et al. 2016.	Poor (< 50%), fair (50–74%) and good (≥75%)	Overall average knowledge. Quarter (25.4%) had good knowledge and about half (49.6%)had fair knowledge about HBV infection
2	Hebo et al. (2019)	Self-administered questionnaire	A set of questions and statements focusing on standard precautions.	Good knowledge or good practice was scored as greater than or equal to 70%	Overall good knowledge on the virus (73.9%) including the transmission and the treatment.
3	Desalegn et al (2013)	Self-administered questionnaire	Questionnaire	Not stated	Overall good knowledge of universal precautions (UPs)
4	Abiola et al. (2015)	Self-administered questionnaire	Pre-tested, structured derived from other similar published studies.	Poor (<50%), fair (50-74%) and good (≥75%)	Overall good knowledge (56.7%) and 43.3% with average knowledge
5	Ngekeng et al. (2018)	Self- administered questionnaire	Questionnaire	Not stated	Overall average knowledge (58.7%)
6	Osagiede et al. (2020)	Self-administered questionnaire	Questionnaire.	< 50% (poor). 50%-65% (fair), > 65% (good)	Overall average knowledge (52.5%)
7	Ijoma et al. (2019)	Self-administered questionnaire	Questionnaire	Not stated	Overall good knowledge on HBV infection (97.0%)
8	Ogundele et al. (2017)	Self-administered questionnaire	Questionnaire	Knowledge scores for individuals were calculated and summed up to give the total knowledge score. A correct response was scored as one point while a wrong answer was scored as zero. The scoring range of the questionnaire was 20 (highest) to 0 (lowest). The mean score was determined and used as cut off. A score below the mean was considered as poor whereas a score above the mean was considered as adequate knowledge about hepatitis	Overall adequate knowledge (61.7%)
9	Oladokun et al. (2021)	Self-administered questionnaire	Questionnaire	Not stated	Overall good knowledge (92.8%)
10	Muhammad et al. (2015)	Self-administered questionnaire	Questionnaire	Not stated	Overall adequate knowledge (58.3%)
11	Akazong et al. (2021)	Self-administered questionnaire	Questionnaire was validated following the guidelines proposed by previous study by Jain et al. Knowledge on the route of HBV transmission was assessed using three questions directly linked with the route of HBV infection.	Adequate knowledge- correct answer was given to all three questions. This minimum of three of three correct answers to define good level of knowledge may seem rigorous. However, this minimum level justified the reasonable difficulty of the questions	Overall average knowledge (67.6%)
12	Rodrigue et al. (2021)	Self-administered questionnaire	Questionnaire	Not stated	Overall, good knowledge (94.7%)

13	Tatsilong et al. (2016)	Self-administered questionnaire	Score based on their responses to 13 questions	A good knowledge was considered as at least 10/13. This minimum of 10/13 defining a good level of knowledge may seem rigorous.	Overall had average knowledge (47.0%)
14	Qin et al. (2018)	Self-administered questionnaire	Questionnaire was developed after reviewing relevant or previous research	Graded the total scores as ‘poor’, ‘intermediate’ or ‘adequate’ level for each study participant based on the distribution located in the tri-sectional quantiles of the grouped data array.	Overall average knowledge (44.1%)
15	Massaquoi et al. (2018)	Self-administered questionnaire	Questionnaire	Not stated	Overall, good knowledge (90.4%)
16	Mangkara et al. (2021)	Self-administered questionnaire	Questionnaire	Not stated	Overall poor knowledge (18.1%)
17	Nguyen et al. (2021)	In depth interview	Mixed-methods study; In depth interview, Questionnaire	Not stated	Overall good knowledge (89.0%)
18	Aziz et al. (2002)	Self-administered questionnaire	Questionnaire	Not stated	Overall good knowledge (90.0%)
19	Memon et al. (2007)	Self-administered questionnaire	Questionnaire	Not stated	Overall poor knowledge (20.0%)
20	Alqahtani et al. (2014)	Self-administered questionnaire	Questionnaire	Not stated	Overall average knowledge (53.0%)
