# Supplementary material for: Seroprevalence of Hepatitis B Among Healthcare Workers in Asia and Africa and Its Association With Their Knowledge and Awareness: A Systematic Review and Meta-Analysis
Source: Front Public Health. 2022 Apr 28;10:859350. doi: 10.3389/fpubh.2022.859350 (PMC9096243; doi:10.3389/fpubh.2022.859350)
Supplement: Supplementary file 4 [file Data_Sheet_4.PDF]

**Supplementary Table 4.** The assessment of awareness level among HCWs.

| No | Author/Year | Awareness assessment | Tool used | Score | Finding |
|---|---|---|---|---|---|
| 1 | Djeriri et al. (2008) | Face-to-face interviews | Questionnaire | Not stated | Overall good awareness (95.0%) |
| 2 | Shao et al. (2018) | Self-administered questionnaire | Questionnaire | Not stated | Overall poor awareness (17.9%) |
| 3 | Anagaw et al (2012) | Interviews and questionnaire | Questionnaire | Not stated | Overall good awareness |
| 4 | Osagiede et al. (2020) | Self-administered questionnaire | Questionnaire | Not stated | Overall good awareness (86.4%) |
| 5 | Ogundele et al. (2017) | Self-administered questionnaire | Questionnaire | Not stated | Overall good awareness (83.7%) |
| 6 | Oladokun et al. (2021) | Self-administered questionnaire | Questionnaire | Not stated | Overall good awareness (92.8%) |
| 7 | Amiwero et al. (2017) | Self-administered questionnaire | Questionnaire | Not stated | Overall good awareness (70.6%) |
| 8 | Mbaawuaga et al. (2019) | Self-administered questionnaire | Questionnaire | Not stated | Overall good awareness (79.6%) |
| 9 | Qin et al. (2018) | Self-administered questionnaire | Questionnaire | Not stated | Overall poor awareness (22.7%) |
| 10 | Ptil et al. (2016) | Self-administered questionnaire | Questionnaire | Not stated | Overall good awareness (98.0%) |
| 11 | Alqahtani et al. (2014) | Self-administered questionnaire | Questionnaire | Not stated | Overall good awareness (99.0%) |
