# Supplementary material for: Seroprevalence of Hepatitis B Among Healthcare Workers in Asia and Africa and Its Association With Their Knowledge and Awareness: A Systematic Review and Meta-Analysis
Source: Front Public Health. 2022 Apr 28;10:859350. doi: 10.3389/fpubh.2022.859350 (PMC9096243; doi:10.3389/fpubh.2022.859350)
Supplement: Supplementary file 6 [file Data_Sheet_6.PDF]

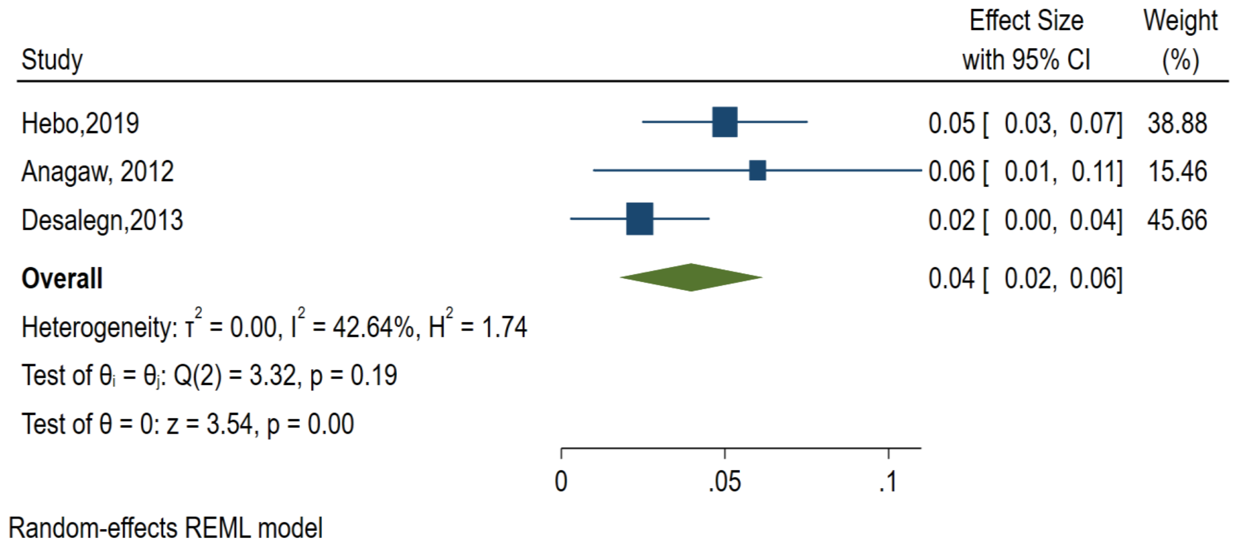

Supplementary Figure 2: Forest plot of seroprevalence estimate in the included studies of the Hepatitis B infection among healthcare workers in Ethiopia.
